# GAPcare: The Geriatric Acute and Post-Acute Fall Prevention Intervention for Emergency Department Patients – A Qualitative Evaluation

**DOI:** 10.21926/obm.geriatr.1904078

**Published:** 2019-10-10

**Authors:** Elizabeth M. Goldberg, Cameron J. Gettel, Kelsey Hayes, Renee R. Shield, Kate M. Guthrie

**Affiliations:** 1. Department of Emergency Medicine, The Warren Alpert Medical School of Brown University, Providence, RI, USA;; 2. Department of Health Services, Policy and Practice, Brown University School of Public Health, 121 South Main Street, Providence, RI, USA;; 3. Department of Emergency Medicine, National Clinician Scholars Program, Yale University School of Medicine, New Haven, CT, USA;; 4. College of Our Lady of the Elms, Chicopee, MA, USA;; 5. Centers for Behavioral and Preventive Medicine, Miriam Hospital, Department of Psychiatry and Human Behavior, The Warren Alpert Medical School of Brown University, Providence, RI, USA;

**Keywords:** Falls, emergency medicine, injury prevention, geriatrics, physical therapy, pharmacist, medication therapy management

## Abstract

**Background:**

Three million US emergency department (ED) visits occur for falls each year. The mortality of falls is increasing and only one fourth of older adults report their fall to their primary care provider, suggesting that valuable preventative opportunities are missed. A fall prevention intervention initiated in the ED immediately after a fall has the potential to reduce subsequent falls, but ED providers lack the time and resources to complete fall risk assessments on their patients. GAPcare, the Geriatric Acute and Post-Acute Fall Prevention Intervention, was developed to address this need.

**Methods:**

GAPcare combines a pharmacist-led medication therapy management intervention with a physical therapist (PT)-administered fall risk assessment and disposition planning. A key objective of this pilot randomized controlled trial (RCT) was to create a patient and caregiver-centric intervention. This manuscript reports on the results of the qualitative companion study in which we conducted in-depth interviews with patients and caregivers to determine their lived experience with the intervention, barriers and perceived impact of the intervention, and to obtain their recommendations for the improvement of GAPcare. We recruited patients and their caregivers from the RCT into 30 minutes interviews in the participants’ home singularly or in dyads (patient and caregiver together). Interviews were audio-recorded, transcribed, and double-coded. We used applied thematic analysis to guide the data analysis.

**Results:**

We conducted 20 interviews; patients (n=12), caregivers (n=11). Patients were on average 83 years old, 7/12 were female, and 2/14 had cognitive impairment. 6/11 caregiver interviews were in reference to a patient with dementia. Patients and caregivers reported they embraced the experience of motivational interviewing elements, citing its collaborative and inclusive nature. Caregivers in particular said they felt that PT helped their loved one recognize and overcome functional limitations. Barriers included lack of time, the burden of coordinating multiple service providers once home, and concerns that PT would be ineffective or increase pain. Areas for improvement included better screening for those who would benefit from the individual components (pharmacy vs. PT consultation), improving identification of GAPcare pharmacists and PTs vs. other hospital staff in the ED, and expanding the role of GAPcare personnel to provide culturally competent, comprehensive care to improve adherence and medication education.

**Conclusions:**

We found that GAPcare, a new team-based intervention for fall prevention in the ED, was welcomed by patients and their caregivers. Several suggestions to improve the intervention were made that will inform the screening, content, and communication with patients in GAPcare.

## Graphical Abstract

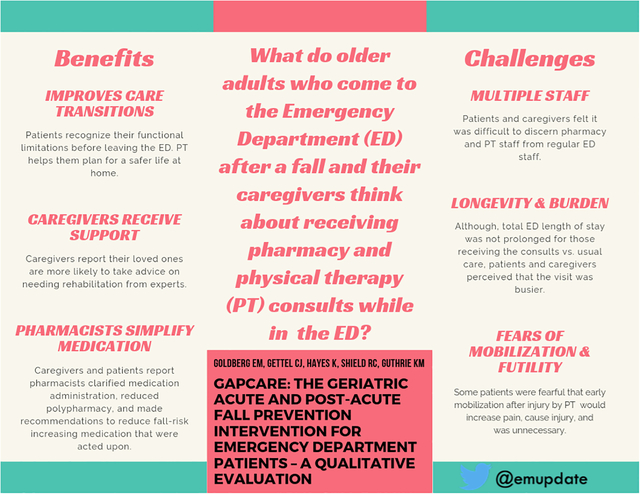

## 1. Introduction

One-third of US Emergency Department (ED) visits for older adults over age 65 are for evaluations after injuries, and falls are the leading fatal and non-fatal reason for injury in older adults [1–3]. Although an interdisciplinary team that includes pharmacists and physical therapists (PTs) who perform standardized fall assessments is a key criterion for US geriatric ED accreditation [4], currently no applicable model exists to guide this care [5]. Further, even though falls are considered one of four high-yield research opportunities in geriatric emergency medicine [6], there is a notable lack of research in EDs evaluating interventions to reduce the occurrence of recurrent falls among older adults.

In response to this need, we designed the GAPcare intervention, which brings together patients, caregivers, pharmacists, PTs, and clinicians to provide a patient-centric, collaborative approach to fall prevention. A lack of patient engagement and uptake of recommendations has been a major drawback of prior fall prevention interventions [7]. Innovative solutions that address these drawbacks are urgently needed, as failure to prevent subsequent falls by older adults results in increased morbidity, mortality [8], healthcare utilization [9], loss of independence [10], and rising health care costs [11]. Stakeholder engagement and co-design with patients are important to creating a successful new care pathway. Trials of multifactorial fall risk assessment followed by referral without assurance of uptake have not been successful [12, 13]. In a recent survey of members of the NEJM Catalyst Insights Council composed of health care executives, clinical leaders, and clinicians, the top four biggest challenges identified in designing patient engagement into effective care delivery processes were the time investment required by the health care team, patient adoption, provider adoption, and cost investment [14].

Qualitative methods have been successfully used to explore these key barriers and discover potential solutions. Through in-depth inquiry, patients and caregivers can become active participants in designing the intervention. Barriers identified in prior qualitative studies to completing fall prevention interventions included the denial of falling risk, the belief that fall prevention was not necessary, and practical barriers to attendance of follow-up [15]. Our aim was to explore these barriers and better understand participants’ lived experience with the intervention, perceived impact of the intervention, and obtain their recommendations for the improvement of GAPcare.

## 2. Materials and Methods

### 2.1 Summary

We collected and analyzed qualitative data using semi-structured interviews of patients and/or their caregivers to assess receptivity and resistance to, and beliefs and perceptions about, incorporating fall prevention into the ED setting through the use of pharmacists and PTs. A formative qualitative study was necessary as this is a new model of care, and we desired to make this care model patient-centric and appropriate for older adults and their unique care needs. We included patients with dementia and their caregivers as they represent an important understudied subgroup of people who experience falls. Qualitative research methods provide an open-ended inquiry to focus on discovery and interpretation, allowing investigators to subsequently tailor ED-based preventative services to older adults. This qualitative study was designed to help explain and interpret the GAPcare quantitative study results, understand the lived experience with GAPcare, uncover actual barriers, and obtain information to improve the intervention.

### 2.2 Participant Recruitment and Setting

We aimed to conduct 20 interviews with patients and/or their caregivers from the intervention arm of the main study for our qualitative evaluation. Recruitment occurred at two different EDs; (1) an academic community hospital and (2) a level I trauma center and tertiary referral ED in the same health system. Individuals 65 and older were eligible to participate in the main study if they presented to the ED within seven days of a fall, could communicate in English or Spanish, and their ED clinician determined they were likely to be discharged from the ED (i.e., not admitted). Patients with cognitive impairment were eligible if a legally authorized representative was present to provide informed consent. Individuals who were altered (e.g., intoxicated), undomiciled, living in a nursing home, or could not provide a phone number for follow-up were excluded. The GAPcare clinical trial protocol [16] and a manuscript detailing the feasibility and acceptability quantitatively include further detail on the study design [17].

Research staff approached individuals who were potentially eligible by electronic health record (EHR) review once they were triaged to a private room. If they consented to participate, they were randomized to the usual care or intervention arm. Participants in the usual care arm received routine care as directed by the ED clinicians. Participants in the intervention arm received the pharmacy and PT GAPcare consultation. GAPcare intervention participants were contacted over the phone and asked to participate in a 30-minute interview. Caregivers were asked to participate in interviews if they were present during the ED index visit. We aimed to complete 20 interviews or interview participants until saturation was reached, as suggested by qualitative researchers, such as Morse [18]. Interviews took place over the phone or in person, depending on the needs of the participant. Dyads were only interviewed in person. When desired by the patient or caregiver, we interviewed them together.

### 2.3 The GAPcare Intervention

In the GAPcare intervention arm, all providers utilized key elements of Motivational Interviewing (MI) to identify aspects of care that required change toward reductions in potential falls and increase motivation [19]. These MI elements included being supportive rather than argumentative, nonjudgmental and collaboratively determining next steps to reduce fall risk. For instance, a pharmacist might say, *“I noticed you are prescribed three medications for pain. Tell me more about what effect this has had on you?”* This kind of question helps to foster a discussion about potential side effects and perceived benefits and drawbacks of being on multiple medications. The following sections describe the pharmacist-led and PT-led parts of the assessment and plan.

#### 2.3.1 Pharmacist-Led Medication Assessment and Plan

Pharmacists completed a brief medication therapy management (MTM) intervention at the intervention participant’s bedside after the ED clinician completed their initial evaluation. Specifically, they asked open-ended questions following key elements of MI (e.g., meeting the person where they are; rolling with resistance; focusing on only the highest impact changes) to determine the participant’s knowledge of their medication regimen and willingness to change medication to reduce fall risk. They also reviewed the medication list obtained by research staff for accuracy using best practices for medication reconciliation [20]; they further discussed medication management with the patient and/or caregiver using MI elements to identify one to three medication that could be stopped or modified to reduce fall risk, and communicated the medication-related action plan in writing to the patient, ED treatment team, and primary care provider (PCP).

#### 2.3.2 PT-Led Fall Risk Assessment and Plan

A PT evaluated the intervention participant at the bedside after diagnostic imaging was reviewed and the ED clinician determined it was safe to mobilize the participant. Specifically, the GAPcare PT performed the following steps: conducted a structured gait, balance, and lower extremity strength assessment (see Table 1), assessed the participant’s ability to function independently on discharge and assisted with discharge planning, and recommended outpatient services/referrals (e.g., home PT). If necessary, they facilitated direct admission to a skilled nursing facility. Then, they communicated the PT action plan in writing and in person to the patient, ED treatment team, and PCP.

### 2.4 Interview Content

We asked open-ended questions of patients and caregivers in the following domains: (1) participant’s experience of the ED screening, treatment, and referrals; (2) receptivity to and concerns about GAPcare; (3) symptom management after ED evaluation; (4) quality of in-ED and outpatient professional and provider communication; (5) participant view of their care transitions; (6) perception of barriers to engaging with follow-up PT care; (7) experiences of clinical trajectories after the ED visit; (8) responses to specific planned components of the intervention, and (9) suggestions to improve GAPcare.

### 2.5 Semi-Structured Interview Procedures

We developed two interview guides – one tailored to the patient and the other to the caregiver. Interview questions were adapted from prior qualitative research studies on older adults and further developed by study authors EG, RS, and KG [15]. The semi-structured interview guide included the study rationale, an overview of the qualitative in-depth interview process and potential queries designed to capture the domains listed above. It contained open-ended questions with follow-up questions and probes specific to study goals. We conducted cognitive testing to test the planned questions and ensure that our questions were understandable and understood as the researchers had intended. Cognitive testing was performed with four older adults prior to recruiting participants into the study. The original study questions were refined based on feedback by these four individuals. Some refined questions incorporated direct quotes of the older adults who did the cognitive testing.

#### 2.5.1 Interviews

Participants were asked to consent to the interview and its recording. The study personnel conducted interviews with patients and caregivers.

#### 2.5.2 Recording

Interviews were recorded, transcribed verbatim, and de-identified. Transcripts were reviewed by the study personnel and corrected when the transcript passage was incomprehensible or erroneous. Recordings were destroyed after completion of the transcription process to ensure confidentiality.

#### 2.5.3 Analysis

We used applied thematic analysis to guide the data analysis [21], which included the following steps: (1) familiarization with the data through reading and re-reading the transcripts and noting initial observations, (2) development of a set of codes based on our interview questions to identify and sort textual data. Four study personnel coded the major topics and sub-topics independently, then reconciled them through team discussion. Coders included the study’s principal investigator (EG) who completed graduate-level coursework in qualitative methods under the tutelage of study authors RS and KG. All coders received coding training and feedback by EG. All transcripts were coded by at least two study personnel. NVivo software (version 12) was used to organize the coded data [22]. After importing the transcripts into NVivo, we entered agreed upon codes into the transcripts. (3) We used a team-based approach and iteratively reduced the data, identified patterns of themes and subthemes that emerged across participants and interviews. (4) We reviewed themes in relation to the coded extracts and the entire dataset and selected representative quotes from the interviews to illustrate the themes. (5) We recorded coding definitions and decisions as well as ideas about emerging themes in an ongoing audit trail [21]. (6) Two study personnel (CG and EG) prepared the analytic narrative and contextualized it using the existing literature.

The hospital Institutional Review Board approved the study. The trial was registered at www.clinicaltrials.gov (ClinicalTrials.gov identifier: NCT03360305).

## 3. Results

### 3.1 Participant Characteristics

The GAPcare intervention recruited 110 patients from January 25, 2018 until March 31, 2019. We held interviews with intervention patients and their caregivers from June 13, 2018 until January 30, 2019. In total, we conducted 20 interviews with 12 patients and 11 caregivers. Three interviews were patient-caregiver dyads. Patients were on average 83 years old, 7/12 were female, and 2/14 had cognitive impairment. 6/11 caregiver interviews were in reference to a patient with dementia. In total, 17/23 participants were women and 6/23 were men. Two patients with cognitive impairment were interviewed, and six of the caregiver interviews were with reference to a patient with cognitive impairment. Of the 11 caregivers, four were daughters, three were wives, one was a sister, one was a niece, one was a female cousin, and one was a son.

We identified five overarching themes from the program evaluation data: (1) experiences with and receptivity to the pharmacy/PT professionals in the ED, (2) barriers to uptake of pharmacy/PT consultation and recommendations, (3) content of the pharmacy/PT consultation, (4) perceived impact of the consultation on the individual, and (5) suggestions for improvement of GAPcare. See Table 1 for an overview of themes and subthemes. Illustrative quotes below are noted with participant characteristics: C, caregiver; CD, caregiver of person with dementia; P, person without dementia; PwD, person with dementia.

### 3.2 Experiences and Receptivity to the Pharmacy and PT Professionals in the ED

#### 3.2.1 Subtheme 1.A

Several participants reported that they felt the pharmacist developed rapport with them, obtained buy-in, and guided them rather than directed them on next steps.

Participants perceived the pharmacist as supportive and described the interaction as a conversation during which their preferences were considered. One caregiver described that their father was taking dandelion tea to treat his bladder cancer. They shared this with the pharmacist and the pharmacist provided them with information on the potential interactions between this tea and another one of the patient’s medications, an anticoagulant, warfarin. The caregiver described that the pharmacist provided additional information about potential interactions with herbal treatments and medication without dismissing the herbal tea as ineffective, a criticism they had heard from prior doctors.

I mean, I’m glad that they’re realizing people are doing things in a holistic way. Instead of criticizing them for being that way, they’re more guiding them on how to still be that way and be safe with the medications they’re taking. That really impressed me because there are some doctors that agree with it, and there are some doctors that think it’s just a crock and get angry. She [the pharmacist] understood that’s what you’re doing, okay, but you need to understand this. So that I think is different, that they went out of their way to explain it like that.

[90C_daughter]

#### 3.2.2 Subtheme 1.B

Patients and caregivers were also receptive to the PT consultations, stating they welcomed feedback on their current mobility and advice on how to improve safety.

One man with a self-described active, independent lifestyle reported positive experiences with the PT intervention, saying, “I have nothing to say, but good things about the experience!” [190P_man]. He recalled being told by the PT that, if anything, he was walking “too fast”. When asked about the GAPcare PT consultation, one caregiver stated, “I thought it was helpful, because it shows you what you can and cannot do, what you are and are not capable of doing on the spot. That, I thought, was good.” [60C_sister].

### 3.3 Barriers to Uptake of Pharmacy/PT Consultation and Recommendations

#### 3.3.1 Subtheme 2.A

Barriers of the GAPcare PT consultation included the fear of mobilization and increasing pain, and prior negative experiences with PT.

Two patients reported that they had pain immediately after the injury and this made them reluctant to engage with PT in the ED and after the ED visit. One patient stated they were apprehensive about accepting the in-ED PT consultation because they experienced an exacerbation of their chronic back pain after the fall and were fearful that physical activity would worsen their pain.

One patient and one caregiver reported that prior negative experiences with PT made them less enthusiastic about the GAPcare PT consultation offered in the ED and afterwards.

I’ve had plenty of it. I can do my own physical therapy. I’m trying to go to [classes at the community club house]. Right now, they’re having a chair yoga [class]. In fact, I’ve had more physical therapy than I have hair on my head. I mean ‘cause I had knee surgery. I had physical therapy then. Then for my back, they tried physical therapy.

[180P_woman].

A caregiver reflected on the PT that was recommended to their cousin while in the ED, *“…and subsequently when she’s been home, she did not want any physical therapy evaluation. No physical therapy. She had a bad experience with it a couple of years ago, and I think maybe that was a cause of the weakness in one foot.”* [140CwD_cousin] It is notable that no patients or caregivers mentioned barriers to uptake of the pharmacy consultation with the exception of time constraints, which are discussed in section 3.5.

### 3.4 Content of the Pharmacy/PT Consultation

#### 3.4.1 Subtheme 3.A

Several caregivers and patients reported the pharmacist explored reasons for medication nonadherence and encouraged that these issues be addressed by the PCP.

For instance, one patient stated the pharmacist delved deeply into reasons for the patient stopping their medication. They uncovered financial concerns and advocated for these concerns to be considered by the PCP. Another patient recalled the pharmacist asked why she stopped a medication that was helping her address urinary incontinence.

Patient: “She said, ‘Well, why did you stop [it]?’ Which I thought was a very good question. I said, ‘Because it was $72 a month.’ She put in the note which I thought was very good to my doctor [to] say, ‘Is there [another medication for urinary incontinence] that would work? I don’t think she missed a trick.”

Interviewer: “She seemed very thorough to you?”

Patient: “Oh, absolutely, in a very nice way. She just really combed right through it.”

[80P_woman]

#### 3.4.2 Subtheme 3.B

Several caregivers, particularly those caring for individuals with dementia, and patients, reported that the pharmacist simplified, improved, and clarified the medication regimen.

One male patient reported the pharmacist decreased the number of pills he needed to take from five pills a day to four, addressing his concerns of being on multiple medications at once. Another caregiver stated that the pharmacist made recommendations to address duplication of medications and stopped medications that may have contributed to the fall. Yet another caregiver of an individual with dementia stated the PCP made changes to the patient’s medication based on the GAPcare pharmacist’s recommendations.

The pharmacist intervention also focused on optimizing the timing of medication administration. One caregiver stated that her husband was confused whether to take medication in the morning or evening and the pharmacist helped them clarify the best time of day to take these medications. Subsequently, the caregiver was able to go through the medications and mark them clearly as morning or evening medication.

Pharmacy consult was fantastic. I thought it was fantastic because a lot of his pills didn’t say ‘give at night’ or ‘give during the day’. And that information was [essential] and then [the pharmacist] sorted out all the pills, told me day or night. I was able to take them and mark them, like ‘p.m.’, ‘a.m.’, it was just great for healthcare.

[100CD_wife]

Another caregiver stated that the pharmacist identified that the time of day the patient was taking the medication may have contributed to the fall.

He was saying maybe her medication should be distributed at a different time during the day so that if she had fallen because of the dizziness then that would make a big difference.

[10C_daughter]

One caregiver of a patient with dementia and Parkinson’s Disease noted that the pharmacist’s suggestion to move the administration of sertraline, a selective serotonin receptor inhibitor for depression, from mornings to evenings helped improve his father’s daytime sleepiness.

The pharmacist recommended changing the times, because the medication made him drowsy, made him kind of unbalanced on his feet. So, we changed a couple of his medications, where he would take them at night instead of in the morning. He’s more alert now during the day more able to move around.

[dyad of 170CD_son&170PwD_man]

Another caregiver noted that the GAPcare pharmacy consult resulted in their father reducing the use of an herbal tea that may have been increasing his risk of bleeding while also on the anticoagulant warfarin. A patient noted that the pharmacist recommended stopping an anticholinergic medication, that was prescribed for sleep, but was not helping the patient sleep better and is also known to increase fall risk. *“I don’t take that amitriptyline anymore at night. It makes me sleep for about an hour and then I’m up all night.”* [160P_woman]

### 3.5 Perceived Impact of the Consultation on the Individual

#### 3.5.1 Subtheme 4.A

Three caregivers thought the in-ED PT intervention helped their family member maintain and improve mobility.

For instance, one caregiver said due to PT her husband was becoming more ambulatory. When she was asked if she found the PT consultation in the ED useful for her husband, she stated, *“Yes, it was. Because right after he was walking very well, and he was doing very well.”* [100CD_wife] Another caregiver reported their mother with dementia had declining mobility, but this stabilized after continued PT. Another caregiver commented that the GAPcare PT was the first to recognize their father had orthostatic hypotension and a balance disorder, which resulted in a referral to a balance specialist, who has since addressed the problem that lead to the fall.

#### 3.5.2 Subtheme 4.B

There was a lack of consensus by caregivers on whether an in-ED PT consult was necessary to ensure the uptake of outpatient PT.

One caregiver suggested that the in-ED PT evaluation prompted PT assessments and therapy in the outpatient setting after ED discharge.

Interviewer: “Did the fall prompt the physical therapy at home?”

Caregiver: “Yes.”

Interviewer: “Is that something that you think he would’ve done even if the physical therapy consult hadn’t happened?”

Caregiver: “No. He wouldn’t have had them come. They only came with discharge.”

[90C_daughter]

On the contrary, one caregiver stated they did not need in-ED PT to know PT after the ED visit was valuable and necessary. She stated it would be helpful for families that may not know of the importance of PT.

The physical therapist was just testing her motor skills and having her stand up and close her eyes, but we were all around her to make sure that she wouldn’t fall. Then she had to walk across the room and walk back. She did well with that. But as far as her closing her eyes, that was a little hairy, but she did well. [After the ED visit]. I called her physical therapist and her physical therapist came in for therapy for a few weeks, and helped with the balance and different exercises for the Parkinson’. I have to say that I would [have involved PT] anyway, but I think it’s good for people who wouldn’t know to get into physical therapy. So, I think it was a good idea, definitely. Absolutely.

[10C_daughter]

#### 3.5.3 Subtheme 4.C

Both patients and caregivers reported that the early recognition of functional limitations led to engagement with care and better ED disposition plans (e.g., hospital admission, ED discharge, SNF placement).

Caregivers reported that the in-ED PT consultation helped their family member recognize their mobility limitations while still in the ED and thus made it easier to convince them that they needed more help than they were previously willing to acknowledge.

And I think that it was beneficial for her, because she needed to see in real time how this has affected [her] movement. And I think she did see that, even though she thought she was okay. From my point of view, I wouldn’t tell her that, “No, you’re not okay.” We had been trying to get her to do [physical therapy] for a while, you know, someone to kind of gauge her gait because there’s been some reason why all of these years you’ve been falling, and she just never addressed it. But I think she needed to see that no, you’re not okay, because I could tell her that all day, and she’s not going to hear it.

[60C_sister]

One patient acknowledged that they did not recognize their lack of mobility after the fall and how hard it would be to cope at home after the fall until the PT mobilized them in the ED.

Then they wouldn’t let me go home. They insisted I go into a nursing home for rehab. The therapist came in and she did like 25 exercises, or something like that, and out of those exercises she did with me, I could only do five. She said, “You can’t go home. Who’s gonna take care of you?” I wouldn’t have just [accepted it otherwise]. I think she’s the one that convinced me to go.

[160P_woman]

A husband-wife dyad discussed that after the fall they made home modifications including moving from an upstairs to a ground level bedroom. They also reported being less reluctant to make modifications to their daily routine and lifestyle to reduce the risk of injury. Yet another caregiver reported that the PT suggested home modifications to reduce future falls and this had a great impact on her mother.

Then when she fell, the physical therapist advised us maybe to get one of those little bars. It goes under the bed, so that when she goes [to] get up, she doesn’t fall out of bed, and that has worked wonders.

[10C_daughter]

### 3.6 Participants Made Various Suggestions for Improvement of the GAPcare Intervention

#### 3.6.1 Subtheme 5.A

Several caregivers felt the length of time of the entire in-ED GAPcare intervention could be shorter and it was easy to get overwhelmed by the many actions and personnel.

Two caregivers reported they were concerned by how long the GAPcare consultation would take, although the content of the intervention was worthwhile.

But the physical therapist came and that was great and the medical feedback, the prescription feedback, but I thought that was all really good, I was glad that I had talked to them. But, I just…[was] concerned about… the longevity of that.

[130C_niece]

Another caregiver reported that she was highly motivated to leave the ED because of the length of time the GAPcare intervention took in addition to the standard medical assessment and treatment. She also reflected on what this may mean for ED managers.

No, because at that point we really weren’t… We were just trying to get out of the hospital, because she had… You know, I don’t think the ER department is wild about [GAPcare] because I think it cuts into their little schedules, they got going and it interrupts their little routine… And, it may have added like two hours to the 10 hour stay that day.

[40CD_daughter]

Another caregiver reported the volume of information provided during the GAPcare consultations may be overwhelming for older persons, who have just had a major healthcare event, and provides suggestions for how the intervention could be improved – including using the teach-back method and relaying information in small quantities.

“I think it’s a lot for someone who [is] older. It is kind of like a lot to take in, because you don’t learn everything in one day. So, don’t forget, now, she still has this swollen face. She also still probably has a headache… She sees your mouth moving, but I’m not sure exactly if she really hears what you’re actually saying. They need to hear. They need to know that you’re there, yes. And they also need to know what it is you do, and that you’re not going to bombard them like, ‘Right now, just want to introduce myself, but if it’s okay with you, I’d like to come back.’ And warn them that it might be two or three chunks of time, because you’ve had your MRI and you’ve had …your blood work and all this other sort of thing, and maybe aska series of questions each time…The other thing is to make sure that they understand what you’re saying. [If] you talk over their head, they’ll miss it. If you talk beneath them or see them as less than someone who deserves this kind of information, you’re going to lose them. And don’t think for a minute they haven’t experienced life enough to recognize that.

[60C_sister]

#### 3.6.2 Subtheme 5.B

Almost one third of patients and caregivers reported problems with distinguishing the GAPcare pharmacist and PT from other ED and outpatient personnel and stated they could not recall which personnel assisted with what change.

One caregiver stated that their aunt had her sleep medication changed, but she could not recall whether the GAPcare pharmacist or PCP prompted this change.

You know, I think actually… they did change the drug that she was taking at night to sleep. I don’t know if that was the reason or if they felt she needed something else. But it did get changed.

[130C_niece]

Three patients and a caregiver stated they did not recall seeing the pharmacist, although on review of the EHR, a consultation was performed and documented in detail. Two women stated they did not meet with PT and a different woman and a man stated that they forgot the content of the discussion with the PT. One man reported he was assessed by PT in the ED and “released”. He recalled being provided with advice on how to fall safely. He stated that he was not advised to continue to engage with PT after his ED visit. However, on review of the PT consultation note, the PT documented that although he was at his “baseline level of function”, he would benefit from home PT. Importantly, this patient returned to the ED with a fall three months later and a new diagnosis of multiple myeloma.

#### 3.6.3 Subtheme 5.C

Two patients without dementia and a daughter of a patient with dementia reported they did not need pharmacy assistance for themselves or their mother.

One female patient stated that the pharmacy consultation must have not been useful because no medication changes were made. Another female patient stated that there was nothing she learned from the interaction. In a further example, the daughter of a patient on hospice stated the pharmacy consultation was less necessary for her mother because the patient’s family had recently had goals of care discussion with their doctor and their medications had just been simplified. This patient’s PCP was a geriatrician. *“We just decided that all that other medication that she was on, at this point in her life [are no longer necessary]. We’re just trying to make her comfortable.”* [40CD_daughter]

#### 3.6.4 Subtheme 5.D

Two patients with chronic progressive mobility impairments reported they did not think PT was beneficial, but they continued it anyway.

One patient with dementia and Parkinson’s Disease reported he felt the PT consult was not necessary and stated he was concerned that he was using a valuable resource others may need. *“But they don’t release me. Why? I question that a lot, because if I’m not needing [it] I don’t want to be taking up time for somebody else. You know what I mean?”* [170PwD_man] However, later in the interview his son stated that his father had still not returned to his normal mobility since the fall and required a lot of help ambulating.

One female patient with myopathy that causes weakness on one side of her body reported a lack of benefit from the in-home PT she received as part of GAPcare after the ED visit. “It’s good exercise, I sit here and do exercises. They don’t help but I do them anyway, keep my legs moving because I can’t stand, and I can’t do a whole lot of walking, because I got a bad back.” [150P_woman] While she did not see the necessity in PT for herself, she did acknowledge that it helped her to be less sedentary.

#### 3.6.5 Subtheme 5.E

One caregiver of an African American GAPcare patient also directed a senior center for low-income adults and made several suggestions to improve the intervention for people of color including: the pharmacist could play a greater role in addressing ED prescribed medication and assessing their cost, could bridge cultural concerns regarding medication compliance, and could fill gaps in medication knowledge and adherence that currently exist between patients and physicians.

I thought [GAPcare] was a pretty good thing simply because she fell, but she didn’t just fall off the turnip truck. She comes with some health issues, you know. Certainly, you don’t want to prescribe something that is going to collide with, for lack of a better word, what she’s already taking. I’m going to share with you something that is typical to this culture - that they talk about - but they’re not going to tell you. That a lot of medication is not designed for people of color. So therefore, you would want to ask as many questions as you possibly can, which they’re going to be afraid to do… They’re not going to ask you that kind of thing, but behind the scenes, that’s what they’re saying. So, you really want, a pharmacist to make sure what [the patient] really knows. I don’t know how you get around that. I really, really don’t, but I’m just sharing with you the kind of things that they say outside of the hospital. So, when you’re prescribing something, all this other stuff has to come into play, even where they live, even what their income might be. Those are the kind of things that guide their mindset. Whether that medication is going to work on someone [of color, like my sister]. Now I went and I picked up her medication, and I’m going to tell you, she didn’t take any of it. Some of the cold packs or whatever it was that they recommended, they were awkward or for whatever reason, she didn’t feel comfortable with it. That was a waste of money.

[60C_sister]

This caregiver highlights the need to provide training to pharmacists on how to address medication choices and adherence in a culturally sensitive way. For instance, it may best to mention at the beginning of the interview that this medication has been shown to be just as effective for people who are White and who are Black and follow up with questions to ask if they have concerns about how they may react to the medication.

#### 3.6.6 Subtheme 5.F

A caregiver and patient reported that they had difficulty completing the GAPcare outpatient care plan because they were already receiving PT for an unrelated problem and another patient stated they were “fired” by their PCP after the ED visit.

The transition of care from the hospital to the community was made difficult for some participants due to lapses in communication and also due to a lack of interoperability between hospital and outpatient EHRs. GAPcare recommendations were provided to the patient and caregiver in writing in addition to the PCP electronically, but patients who lacked PCP follow-up may have missed opportunities to have their recommendations acted upon. This was likely to occur if the PCP that received the GAPcare recommendations did not provide continued care.

## 4. Discussion

Our semi-structured interviews with 23 patients and caregivers who had recently experienced a new model of care in the ED revealed important insights into the potential impact of an ED-specific multidisciplinary fall prevention team, as well as barriers and areas for improvement. The pharmacist MTM session was well regarded and caregivers enjoyed the MI approach rather than being directed when and how to take their medication. Many participants reported the pharmacist consultation helped them clarify and simplify their medication regimen. This is important because polypharmacy and medication administration errors increase fall risk. Similarly, the PT consultation helped patients recognize post-injury functional limitations and helped them develop strategies to improve their mobility, home environment, and adopt assistive devices.

Although MTM has been proven to be beneficial in reducing fall-risk increasing medication [23] and PT is effective at preventing fall-related ED visits [24], some patients may not garner a benefit from both consultations. Given resource and time constraints, a future version of GAPcare could investigate how to screen patients that would particularly benefit from each evaluation. However, as the qualitative interviews demonstrated, many participants had perceptions that they would not benefit from either the PT or pharmacy consultation, but once they received the intervention their perception changed. “You don’t know, what you don’t know” certainly applies to patients’ typical responses to fall prevention; many older adults do not want to acknowledge the fall as a sentinel event worth intervening upon. The lack of fall-related prevention knowledge, the stigma of falls, and the higher likelihood of cognitive impairment in this population make delivering interventions particularly challenging.

Most research into in-ED fall prevention interventions has focused on creating a screening tool [25, 26] to help clinicians target only the most high-risk patients for fall prevention efforts. The GAPcare approach is to intervene on every patient presenting after a fall. The American Geriatric Society recommends that every patient seeking ED care after a fall receives a falls risk assessment and only 1 in 4 patients report their falls to their PCPs [27]. Therefore, fall prevention efforts not started in the ED may never be initiated. The all-comers approach may be preferred over instituting a screening tool for many reasons. ED nurses and clinicians already perform mandated screenings for many illnesses[5] and instituting new screening tools to determine the benefit of in-ED pharmacy and PT consultations for those presenting after a fall are unlikely to be widely adopted. An additional difficulty with a screening tool is that in this case the professional performing the consultation is best suited to perform the screening. This is because ED nurses and doctors often do not have the time or expertise to perform a comprehensive medication reconciliation to predict which patients could benefit from a pharmacy or PT consultation.

Patients and caregivers voiced concerns over PT lacking effectiveness for other conditions for which they had prior PT treatment, potentially increasing pain, and being difficult to coordinate out of the hospital. However, patients and caregivers recognized that PT could help patients recognize mobility limitations and the need for skilled nursing placement, a new assistive device, or future PT. Future versions of GAPcare could better integrate MI elements into the PT consultation to help patients recognize new mobility limitations resulting from the fall and the potential mismatch between their available home resources and post-injury needs.

Overall, as with any new model of care, attention needs to be turned towards ensuring participants understand the components and the necessary time commitment. The accompanying quantitative analysis of GAPcare found that each individual consultation took on average 20 minutes to complete, and the consultation did not increase ED length of stay (LOS) for participants in the intervention arm compared to those in the usual care arm [17]. Branding the intervention, assigning uniforms, and instituting frequent reminders and prompts in the post-ED follow-up period, may aid with the identification of personnel and decrease the sense many had of being overwhelmed by ED care and GAPcare follow-up components. Many older adults have multiple care providers and healthcare appointments; clearly distinguishing the GAPcare intervention from usual care could help individuals better communicate with their PCP and other healthcare providers. This could benefit care transitions and intervention uptake.

Our findings that older adults are concerned about further injury if PT is offered too early are important, but early post-injury PT may also improve recovery and could reduce the fear of falling [28]. Any in-ED fall prevention intervention will need to address these concerns and provide information about the safety of PT. Prior studies have shown that the typical ED management of adults who present after a fall focuses on injury assessment with little consideration of reasons for the fall and no provision of fall prevention resources on discharge [29]. PTs are experienced at providing patient education surrounding safety awareness, mobility, and performing fall risk assessments. Prior research has shown ED-based PT is effective in reducing falls for high risk patients who present to EDs [30]. ED-based PT services are also embraced as helpful by ED physicians [31].

### 4.1 Limitations and Future Directions

Although our study findings will be essential for enhancing the GAPcare intervention, we did not interview ED clinicians and PCPs. Their perspectives are also be valuable in improving the intervention and we expect to discover more barriers and potential solutions in the planned additional interviews of these groups. Additionally, our participants represent a cohort of older adults and their caregivers that agreed to participate in interviews and the GAPcare intervention and their responses may be different than patients who declined participation. Additionally, interviews were conducted at varying times after the initial ED visit and in this group of patients recall may be limited. However, research shows that patients with dementia are consistent with their preferences, and we feel it is important to include their opinions. Finally, in order to best accommodate mostly frail older adults and their caregivers the interview settings varied; some were over the phone, some in-person, some in dyads. This may have had an effect on the data analysis but was preferable to not capturing these opinions.

## 5. Conclusions

Most older adults and their caregivers interviewed on their ED care experiences with GAPcare and recovery after a fall reported that they valued in-ED pharmacy and PT consultation. Our sample reported they made changes to their medication, home environment, and routine activities as a result of these consultations. However, they noted that they often could not distinguish between the multiple staff they met during the ED visit and the longevity of the intervention was a concern. Stakeholders should ensure that education is provided to assuage concerns of worsened pain, prolonged ED stay, and highlight the potential benefits of early rehabilitation and medication changes in preventing future falls. Overall, participants embraced the MI elements employed by consultants and GAPcare could serve as a model for ED-initiated fall prevention efforts to provide patient and caregiver-centric care to the millions of Americans seeking emergency care after a fall.

## Figures and Tables

**Table 1:** Overview of themes and subthemes.

Theme	Subtheme
**Experience and Receptivity to the Pharmacy and PT Professionals in the ED**	Several participants reported they felt the pharmacist developed rapport with them.
	Patients and caregivers were also receptive to the PT consultations, stating they welcomed the feedback on their current mobility and advice on how to improve safety.
**Barriers to Uptake of Pharmacy/PT Consultation and Recommendations**	Barriers of the GAPcare PT consultation included the fear of mobilization and increasing pain, and prior negative experiences with PT.
**Content of the Pharmacy/PT Consultation**	Several caregivers and patients reported the pharmacist explored reasons for medication nonadherence and encouraged that these issues be addressed by the PCP.
	Several caregivers, particularly those caring for individuals with dementia, and patients, reported that the pharmacist simplified, improved, and clarified the medication regimen.
**Perceived Impact of the Consultation on the Individual**	Three caregivers thought the in-ED PT intervention helped their family member maintain and improve mobility.
	There was a lack of consensus by caregivers on whether an in-ED PT consult was necessary to ensure the uptake of outpatient PT.
	Both patients and caregivers reported that the early recognition of functional limitations led to engagement with care and better ED disposition plans (e.g., hospital admission, ED discharge, SNF placement).
**Participants Made Various Suggestions for Improvement of the Gapcare Intervention**	Several caregivers felt the length of time of the entire in-ED GAPcare intervention could be shorter and it was easy to get overwhelmed by the many actions and personnel.
	Almost one third of patients and caregivers reported problems with distinguishing the GAPcare pharmacist and PT from other ED and outpatient personnel and stated they could not recall which personnel assisted with what change.
	Two patients without dementia and a daughter of a patient with dementia reported they did not need pharmacy assistance for themselves or their mother.
	Two patients with chronic progressive mobility impairments reported they did not think PT was beneficial, but they continued it anyway.
	One caregiver of an African American GAPcare patient made several suggestions to improve the intervention for people of color.
	A caregiver and patient reported that they had difficulty completing the GAPcare outpatient care plan because they were already receiving PT for an unrelated problem and another patient stated they were “fired” by their PCP after the ED visit.
